# Preventive peer-educational activities: what can medical students do to potentially save lives?

**DOI:** 10.1186/1757-7241-21-S1-S24

**Published:** 2013-05-28

**Authors:** P Abreu-Reis, A Nasr, B Scheffer, F Tomasich, I Collaço, M Manfrinato, J Cruz, H Slongo, L Bordingon

**Affiliations:** 1 Médico pela UFPR - Turma 134. Brazil

## Introduction and objectives

Traffic injuries are amongst the main causes of death worldwide. Even with the advances in technology, there are still 50% of deaths that cannot be reduced by medical care improvement. These important injuries can though be reduced by prevention of trauma. One of the best ways to address this issue is through incorporating preventive measures to the daily routine of schools. It is the aim of this study to assess children’s perception on traumatic events and to introduce a cost-effective peer-education preventive action.

## Methods

A prospective interventional comparative study with children from a basic school in south Brazil. A questionnaire with 9 decision-making questions about traffic scenarios was applied by volunteer medical students from May to June 2012, before and after a peer-educational lecture on prevention of traffic injuries. There were 20 epidemiological questions as well. Data collected was compared between the pre and the post-tests of the same students. Statistical analysis was performed using the chi-square for discrete, and the students’ t-test for continuous variables.

## Results

246 students answered the questionnaires. The mean age was 10.19 years old. 21% reported that always crosses the street alone, while 47% only cross with an adult. Most of the students said they always cross streets in the zebra-crossing and look to both sides before crossing (60.71% and 84.9%, respectively). Impressively, 12.55% said they often/sometimes drive a car or a motorcycle. Also, 30.76% ride a bike in between the cars. Furthermore, 77.48% of the students use the front seat of the car. Regarding safety issues, only one third have a horn in their bikes, and less than half use helmet when playing. When comparing their assessments, there was a higher number of correct answers in the decision-making section in the post-test (5.21vs3.93, p=0.000001161). The estimated overall cost was 20 dollars.

## Conclusion

Preventive measures urge to be incorporated to schools curricula. Peer educational actions are a cost-effective for spreading medical knowledge amongst children and youth.

